# Post-bottleneck increase in mitochondrial DNA diversity in Yaku sika deer (*Cervus nippon yakushimae*) on Yakushima Island, Japan

**DOI:** 10.1038/s41598-025-23191-9

**Published:** 2025-11-12

**Authors:** Yoshimi Agetsuma-Yanagihara, Takashi Hayakawa, Naoki Agetsuma

**Affiliations:** 1 Waku Doki Science Planning, Sapporo, Hokkaido 065-0016 Japan; 2 Faculty of Environmental Earth Science, Hokkaido University, Sapporo, Hokkaido 060-0810 Japan; 3 Field Science Center for Northern Biosphere, Hokkaido University, Sapporo, Hokkaido 060-0809 Japan

**Keywords:** Geographical isolation, Haplotype network, Noninvasive method, Pyroclastic sediment, Simultaneous dispersal, World Natural Heritage Site, Ecology, Ecology, Evolution, Genetics

## Abstract

We investigated the genetic diversity and divergence of sika deer (*Cervus nippon*) descended from survivors of a catastrophic pyroclastic flow event approximately 7,300 years ago by analyzing the 894 base pairs mitochondrial DNA control region extracted from fecal samples. We focused on Yaku sika deer (*C. n. yakushimae*) across the remote islands Yakushima and Kuchinoerabujima in southern Japan. On Yakushima, 18 haplotypes were detected, with significant regional variation. Two subpopulation structures on Yakushima aligned with the distribution of pyroclastic flow deposits. Two haplotypes were found on Kuchinoerabujima, one shared with Yakushima. Reconstructed haplotype networks with sika deer from various regions of the Japanese Archipelago suggested that a historical simultaneous dispersal event occurred in the Yakushima population. The Yakushima and Tanegashima populations (*C. n. mageshimae*) formed their own clusters and were clearly separated from other southwestern Japanese populations. The lack of common haplotypes between these populations suggests that the Yakushima population experienced sufficient generational changes for genetic drift to fix region-specific mutations after geographical isolation approximately 37,500 years ago. The high genetic diversity of the current Yakushima population may have been acquired through simultaneous dispersal approximately 4,900–6,500 years ago after the pyroclastic flow-induced population bottleneck approximately 7,300 years ago.

**Supplementary Information:**

The online version contains supplementary material available at 10.1038/s41598-025-23191-9.

## Introduction

Genetic diversity is one of the most important factors for the conservation of wild animal populations because low genetic diversity leads to decreased population fitness^1^ and may even result in vulnerability to extinction by factors such as inbreeding and loss of adaptability to environmental changes^2,3^. Population bottlenecks, i.e., extreme reductions in population size, have occurred in various populations, often leading to a decrease in genetic diversity^4–6^. Understanding the recovery process of genetic diversity after bottlenecks will help conserve populations and provide insights on the formation of local genetic structures and species speciation. In this study, we analyzed the genetic diversity of an isolated population of sika deer (*Cervus nippon*) that likely experienced a bottleneck due to catastrophic events and examined when and how its genetic diversity was acquired.

**Fig. 1:**
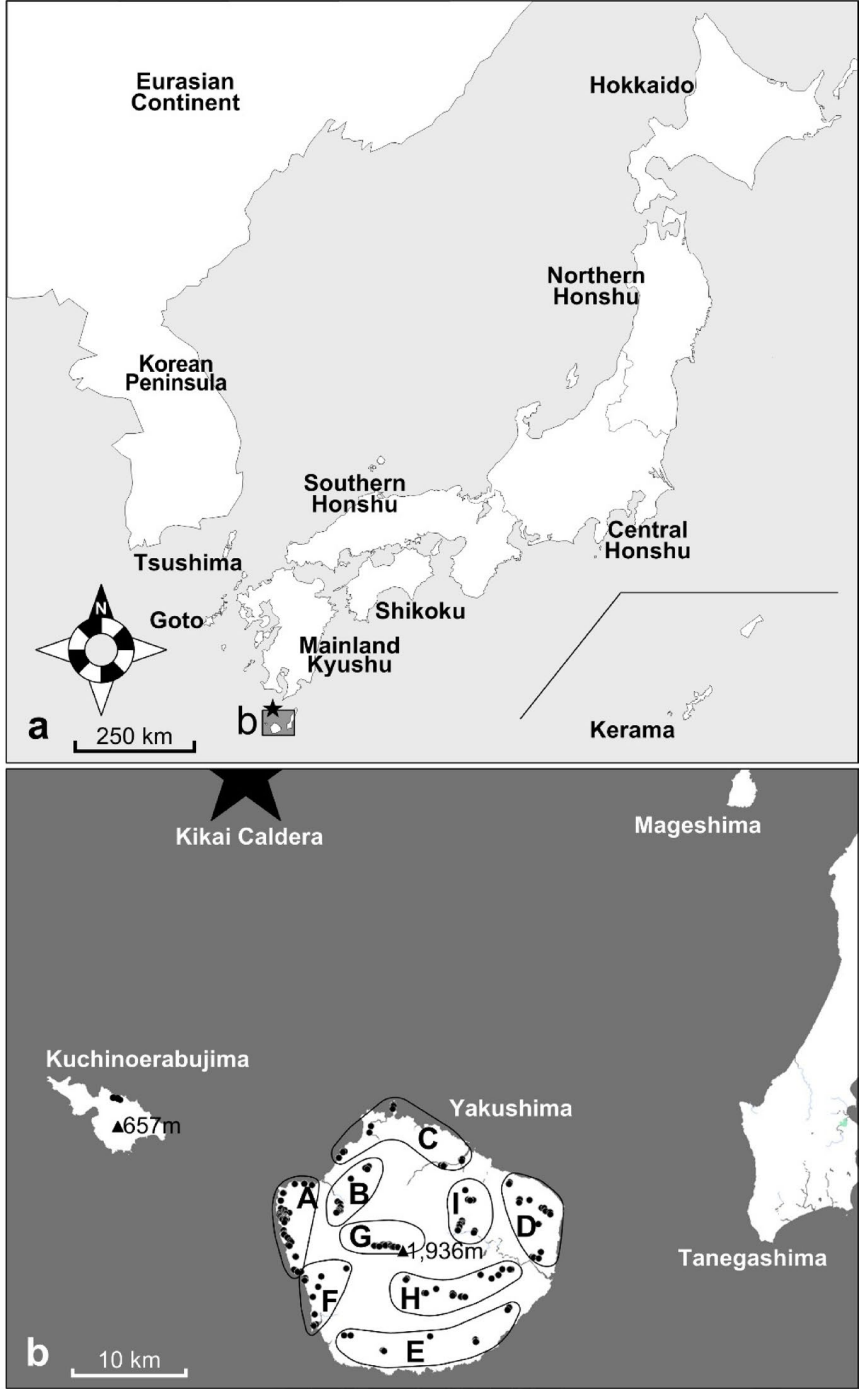
Study site, sample collection points, and area divisions used in our analysis. (**a**) Map of the Japanese Archipelago and the locality used for haplotype network analysis. The star (★) represents the Kikai Caldera. An enlarged view of the area enclosed by the solid line is shown in “b”. (**b**) Enlarged map of Yakushima and Kuchinoerabujima. Black circles (●) represent the sample collection points. The black triangles (▲) represent the highest elevation points on each island. The A–I areas used in the analysis are circled by solid lines. This Figure was created based on the GSI Map Vector of the Geospatial Information Authority of Japan (GSI) (https://maps.gsi.go.jp/vector/#7/36.104611/140.084556/&ls=vstd&disp=1), using the GSI Map Sheet Tool (Ver. 2.0.0.1) provided by GSI.

Sika deer are widely distributed in East Asia, extending from central China (western end) to Japan (eastern end) and from the easternmost part of Russia (the northern end) to southern China and Vietnam (southern end); however, most of the continent’s wild populations are extinct or threatened today^7^. In contrast, sika deer populations in the Japanese Archipelago temporarily declined due to habitat destruction during the modern and contemporary periods, but have been recovering since the 1970s^8–10^. The sika deer populations across the Japanese Archipelago are classified into six subspecies in each habitat (Fig. 1a,b): *C. n. yesoensis* (Hokkaido; 77,984 km^2^), *C. n. centralis* (Honshu; 227,943 km^2^ and Tsushima Islands), *C. n. nippon* (Shikoku; 18,298 km^2^, Mainland Kyushu; 36,783 km^2^ and Goto Islands), *C. n. mageshimae* (Tanegashima; 445 km^2^ and Mageshima; 8 km^2^), *C. n. yakushimae* (Yakushima; 504 km^2^ and Kuchinoerabujima; 36 km^2^), and *C. n. keramae* (Kerama Islands)^11^. However, studies based on mitochondrial DNA (mtDNA)^12–14^ and nuclear DNA microsatellite analyses^15^ have reported the existence of two distinct lineages—northern and southern—highlighting the need to elucidate genetic structure as a basis for defining conservation management units^16–18^. These lineage boundaries are consistently recognized in Southern Honshu and Shikoku^12–15^, with divergence times estimated at approximately 0.35 million years ago (mya) based on mtDNA D-loop partial sequences^12^, 0.41 mya based on complete cytochrome b sequences^19^, and 0.53 mya based on mitogenomic data^20^.Furthermore, sika deer populations in Japan exhibit karyotype polymorphisms (2n = 64, 66, or 68), and their hybrids (2n = 65 and 67) have been identified^21–23^. According to Harada^21^, the northern lineage has 2n = 68 chromosomes, which is the oldest and most widely distributed on Hokkaido, Northern Honshu, most of Central Honshu, and Shikoku (Fig. 1a). For the southern lineage, most Southern Honshu populations have 2n = 66 chromosomes, while most Kyushu populations have 2n = 64 chromosomes.

The Yaku sika deer (*C. n. yakushimae*) examined in this study is a subspecies of sika deer and is distributed exclusively on Yakushima and Kuchinoerabujima Islands^24^. Although the Yaku sika population was clustered with the southern Japanese lineage based on mtDNA^14,25^ and nuclear DNA phylogeny^15^, the genetic distance was significantly different from those of the other populations of the southern Japanese lineage^14,15,25,26^. Polymorphisms of Y chromosome simple sequence repeats (SSRs) was also clearly differentiated from those of the other Japanese populations^18,20^. The Yakushima populations have 2n = 68 chromosomes, which is similar to that of the northern lineage populations. Thus, the karyotype characteristics of the Yakushima population could not be classified simply based on geographical location. The Yaku sika deer is the smallest among the subspecies of the Japanese sika deer and has shorter limbs; these morphological traits are thought to be due to genetic characteristics^20,26–28^. These deer are thought to have evolved in Yakushima, an island that is not inhabited by medium- and large-sized carnivorous predators^29^. Miniaturization and limb shortening are typical morphological traits that frequently evolved in ungulates inhabiting small islands without predators^30^. In addition to these morphological features, the ecology^31–33^ and life history^34^ of these deer clearly differed from those of other sika deer subspecies. In the western lowland of Yakushima, females give birth for the first time at 3–5 years of age, and three-pointed antlers appear in males aged 4–5 years or older (Agetsuma and Agetsuma-Yanagihara unpublished data). The average body weight of females and males older than 4 years is 21 kg and 28 kg, respectively (Agetsuma and Agetsuma-Yanagihara unpublished data). The mean annual home range size (expressed as a 90% fixed kernel) for females and males aged more than 4 years is 12 ha and 36 ha, respectively, which is smaller than that of other subspecies^35^.

Approximately 7,300 years ago, the Yaku sika deer population would have encountered catastrophic damage due to the eruption of a submarine volcano named the Kikai Caldera (Fig. 1a,b). Pyroclastic flow from the Kikai Caldera (Koya pyroclastic flow) ran across the islands and deposited sediments over most parts of Yakushima and Kuchinoerabujima^36^. Wildlife populations in these islands were greatly impacted by the pyroclastic flow. Although migratory individuals from different populations generally bring diversity to genetically impoverished populations, this requires connectivity between habitats that allows for movement between populations. However, for populations on isolated islands, recovery of genetic diversity is more difficult. In fact, the sympatric Yakushima macaques (*Macaca fuscata yakui*) is thought to have experienced a strong bottleneck after the catastrophe^37^. Thus, sympatric macaques and Yaku sika deer might have been similarly impacted. Nonetheless, Terada et al.^17^ performed a microsatellite genetic structure analysis and showed that Yaku sika deer have high genetic diversity despite the small island population and are separated into two or four subpopulations (northern and southern, or northern, southern, eastern, and western parts of Yakushima); however, sufficient samples from the wildlife protection areas and mountainous areas were not analyzed. We examined whether the Yaku sika deer experienced a bottleneck, and if so, how and when the current genetic diversity was acquired. Our analyses will help reveal how genetic structure and diversity are reformed in isolated populations. mtDNA is a better marker for detecting genetic variation within a single species than nuclear DNA because it does not involve recombination and mutations accumulate rapidly due to the high rate of base substitution^38,39^. Thus, mtDNA is suitable for investigating regional differences within small islands such as Yakushima. Therefore, by examining the mtDNA of deer from whole areas of Yakushima Island, we investigated the process by which genetic diversity was obtained in the island population.

## Results

### Genetic relationships among populations from different areas of Yakushima

**Fig. 2:**
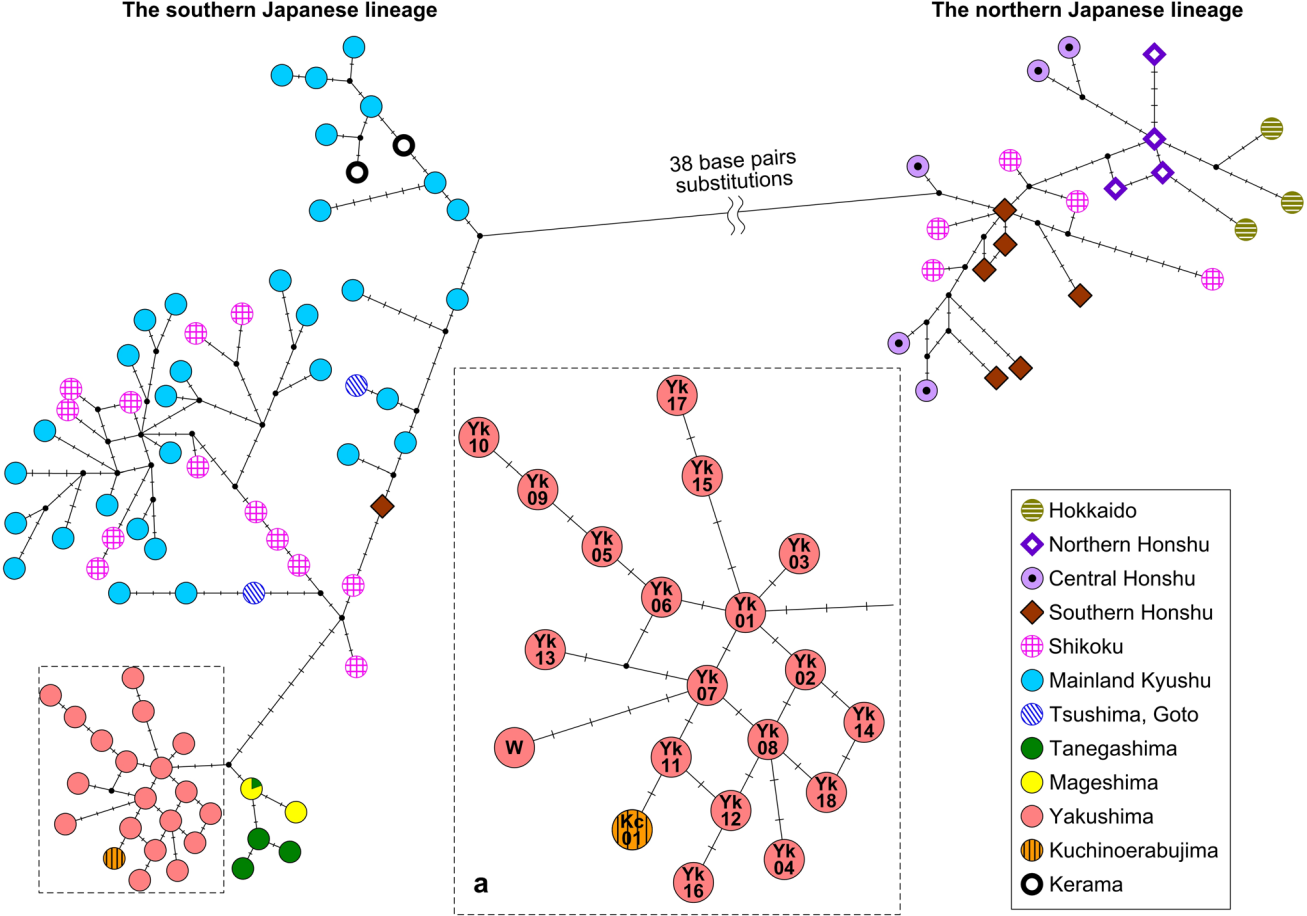
Haplotype network based on the mitochondrial DNA control region of sika deer (*Cervus nippon*) in the Japanese Archipelago. We analyzed 110 haplotypes (Supplementary Table S1) by combing data from the NCBI registry and this study. Yaku sika deer haplotypes are enclosed in dashed boxes and enlarged Figures are shown in a separate box “a”; Yk01–Yk18 and Kc01 represent the haplotype names in this study, and W represents the sequence (AB218689) from Wada et al.^40^. The dashes in the branches represent mutation points and the small black circles represent hypothetical haplotypes. Created using Network 10.2.0.0 (https://www.fluxus-engineering.com/sharenet.htm).

We determined the partial mtDNA control region sequence in 380 out of 419 samples in Yakushima and 2 out of 5 samples in Kuchinoerabujima. A total of 303 samples from Yakushima and 1 sample from Kuchinoerabujima yielded more than 99% of the 894 base pairs (bp) partial sequence, beginning at the 48th bp of the reference sequence registered in the NCBI database (Accession No. AB279718.1; https://www.ncbi.nlm.nih.gov). This 894 bp partial sequence of the mtDNA control region in Yaku sika deer contained 13 single-nucleotide substitutions (all transitions) and two single-nucleotide length polymorphisms. By combining these data, 19 haplotypes were identified from Yakushima and Kuchinoerabujima (Supplementary Table S1 online). The other sample from Kuchinoerabujima, Kc02, yielded a slightly shorter sequence of 841 bp, but it was identical to the Yakushima haplotype Yk06 within the aligned region (Supplementary Table S1 online). A total of 20 haplotypes, including Kc02, were deposited in the DNA Data Bank of Japan (DDBJ; https://www.ddbj.nig.ac.jp/index.html) under Accession Nos. LC664029–LC664048. Due to its shorter sequence length, Kc02 was excluded from subsequent analyses. A haplotype network constructed using the haplotypes obtained in this study, together with the sequence registered in NCBI (AB218689) from Wada et al.^40^, exhibited a radial shape centered on Yk01, with most haplotypes connected by a single mutation (Fig. 2a).

Among the 303 samples from Yakushima, the total haplotype diversity (*h*) was 0.9006, with the diversity from each area in Yakushima ranging from 0.6931 (Area H) to 0.8480 (Area F). The total nucleotide diversity (*π*) was 0.00293, with the diversity from each area ranging from 0.00063 (Area H) to 0.00330 (Area C) (Table 1). Yk01, Yk05, Yk06, Yk07, and Yk11 were distributed over a wide area of the island, Yk09 was distributed only in the central to northern part of the island (areas B, C, G, and I), while Yk04 was primarily (96.5%) distributed in Area A (Supplementary Table S2 online).

**Table 1:** Summary statistics of nucleotide sequence data calculated based on the mitochondrial DNA control region 894 base pairs of Yaku sika deer (*Cervus nippon yakushimae*) in Yakushima calculated using Arlequin ver 3.5.2^93^.

Area	No. of samples N	No. of polymorphic sites S	No. of haplotypes H	Haplotype diversity h	Nucleotide diversity π	Mean No. of differences k
A	104	10	8	0.6953	0.00277	2.3373
B	18	6	4	0.7386	0.00266	2.2422
C	22	9	7	0.8355	0.00330	2.7797
D	30	5	6	0.8299	0.00194	1.6349
E	21	6	6	0.7667	0.00125	1.0526
F	19	6	7	0.8480	0.00167	1.4090
G	38	8	6	0.7952	0.00240	2.0279
H	28	5	6	0.6931	0.00063	0.5313
I	23	6	6	0.6996	0.00218	1.8337
Northern: B/C/G/I	202	13	15	0.8644	0.00247	2.0856
Southern: A/D/E/F/H	101	12	11	0.8323	0.00266	2.2465
Total	303	14	18	0.9006	0.00293	2.4732

**Fig. 3:**
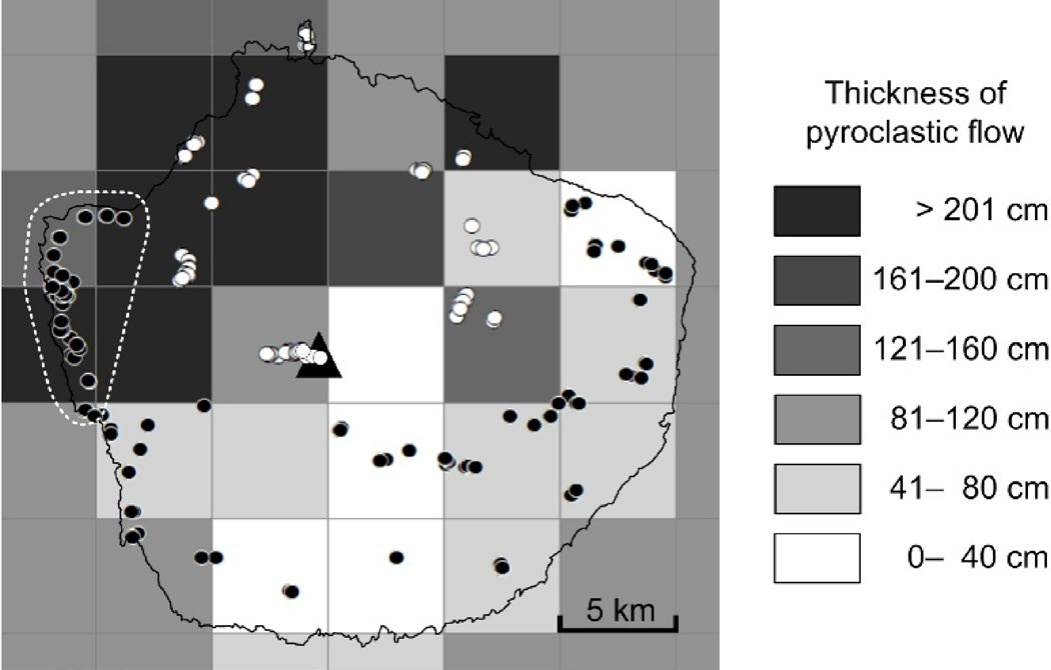
Genetic population structure of Yaku sika deer (*Cervus nippon yakushimae*) and thickness of the pyroclastic flow deposited on Yakushima. Black triangle (▲) represents the highest elevation point (1,936 m asl). Thickness of pyroclastic flow deposits estimated by spatial interpolation (Gaussian model, 5 km mesh) based on Geshi^36^. SAMOVA ver. 2.0^92^ was used to divide the Yaku sika deer into two genetic populations, i.e., north (open circles ○; areas B/C/G/I) and the other areas (filled circles ●; areas A/D/E/F/H: Fig. 1b). However, Area A, surrounded by the dashed line, did not belong to the northern group, even though it was heavily affected by the pyroclastic flow.

With respect to the relationship between geographical location and genetic differentiation, spatial analysis of molecular variance (SAMOVA) revealed a fixed index *F*_CT_ = 0.2250 (*p* = 0.00587) when the number of groups *K* equaled 2 (i.e., northern [Areas B, C, G, and I] and southern populations [Areas A, D, E, F, and H]). At *K* = 3, Area A was distinguished from the southern population and became a single group. At *K* = 4 and 5, Area A remained the lone group. The northern population was located on the thick sediment of the Koya pyroclastic flow, whereas the southern population (excluding Area A) was on the thin sediment (Fig. 3). Among areas of the southern population, Area A had exceptionally thick sediment. Genetic differentiation between northern and southern populations examined by analyses of molecular variance (AMOVA) indicated that 26.27% of the variation in the total sample population was due to the differences between areas (*F*_ST_ = 0.26267, *p* < 0.0001, Supplementary Table S3 online). In addition, pairwise *F*_ST_ values between areas showed significant differentiation among many areas. Especially, the *F*_ST_ of the population in Area A was significantly different from that of all the other eight areas (*p* < 0.001; Table 2).

**Table 2:** Degree of genetic differentiation among nine areas (A–I) by pairwise *F*_ST_ values based on the mitochondrial DNA control region 894 base pairs.

Area	A	B	C	D	E	F	G	H
B	0.30841*							
C	0.22567*	0.01147						
D	0.16674*	0.14218	0.10766*					
E	0.20969*	0.31589*	0.27109*	0.05704				
F	0.21569*	0.11462	0.08880	0.04066	0.23808*			
G	0.34755*	0.07034	0.04993	0.25550*	0.43062*	0.14424		
H	0.23864*	0.47526*	0.38588*	0.16329*	0.05606	0.39259*	0.52454*	
I	0.35317*	−0.00892	0.03930	0.24208*	0.43852*	0.16998*	0.02683	0.56396*

*Significant differences after Bonferroni correction (*p < 0.001)

### Demography of Yaku sika deer population on Yakushima Island

**Table 3:** Tajima’s *D*, Fu’s *F*s, Raggedness index *r*, and Sum of squared deviation (SSD) for southern and northern populations based on the mitochondrial DNA control region 894 base pairs.

Area	Tajima’s *D*		Fu’s *F*s		Raggedness index		SSD	
	*D*	p value	*F*s	p value	*r*	p value	*SSD*	p value
Northern: B/C/G/I	0.1395	0.634	−0.4936	0.471	0.0242	0.870	0.0082	0.690
Southern: A/D/E/F/H	0.2591	0.661	−1.4767	0.363	0.0707	0.010	0.0192	0.010
Whole	0.6685	0.783	−1.6702	0.338	0.0259	0.250	0.0041	0.100

**Fig. 4:**
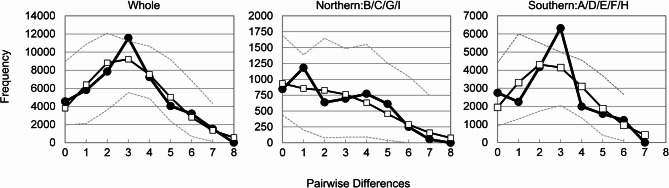
Mismatch distribution of Yaku sika deer (*Cervus nippon yakushimae*) based on the mitochondrial DNA control region 894 base pairs. The thick solid line represents the observed mismatch distribution (—●—). The thin solid line represents the demographic expansion model (—□— ). Two dashed lines indicate 95% confidence intervals (----).

The values of Tajima’s *D* and Fu’s *F*s from 1,000 repeats with coalescent simulations were 0.6685 (*p* = 0.783) and −1.6702 (*p* = 0.338) for the whole population, 0.1395 (*p* = 0.634) and −0.4936 (*p* = 0.471) for the northern population, and 0.2591 (*p* = 0.661) and −1.4767 (*p* = 0.363) for the southern population, with none reaching statistical significance (Table 3). Mismatch analysis showed that the whole population and southern population had a unimodal distribution with a peak at three substitutions (Fig. 4 left and right), while the northern population showed a bimodal distribution with peak at one and four substitutions (Fig. 4 center). The sum of squared deviations (SSD) statistic and raggedness index for the whole population and northern population indicated that the observed frequency distribution fit the distribution expected under the demographic expansion model (SSD = 0.0041 and 0.0082, *p* = 0.100 and 0.690, raggedness index *r* = 0.0259 and 0.0242, *p* = 0.250 and 0.870; Table 3); however, it was rejected for the southern population (SSD = 0.0192, *p* = 0.010, raggedness index *r* = 0.0707, *p* = 0.010; Table 3).

### Haplotype network and molecular phylogenetic tree of Japanese sika deer

The haplotype network diagram drawn using the Yaku sika deer sequences obtained in this study and the 91 sika deer sequences (Supplementary Table S4 online) registered at the NCBI showed that the populations in Japan could be clearly divided into two lineages, north and south, which is similar to the findings of previous studies (Fig. 2). The Yakushima and Kuchinoerabujima populations formed a single cluster at the edge of the southern lineage, with the Tanegashima and Mageshima populations located nearby and the Mainland Kyushu, Tsushima/Goto, Kerama, Shikoku, Honshu, and Hokkaido (Fig. 1a,b) populations located more distally (Fig. 2). All populations west of Mainland Kyushu were included in the southern lineage, while the Shikoku and Honshu populations were divided into both northern and southern lineages.

**Fig. 5:**
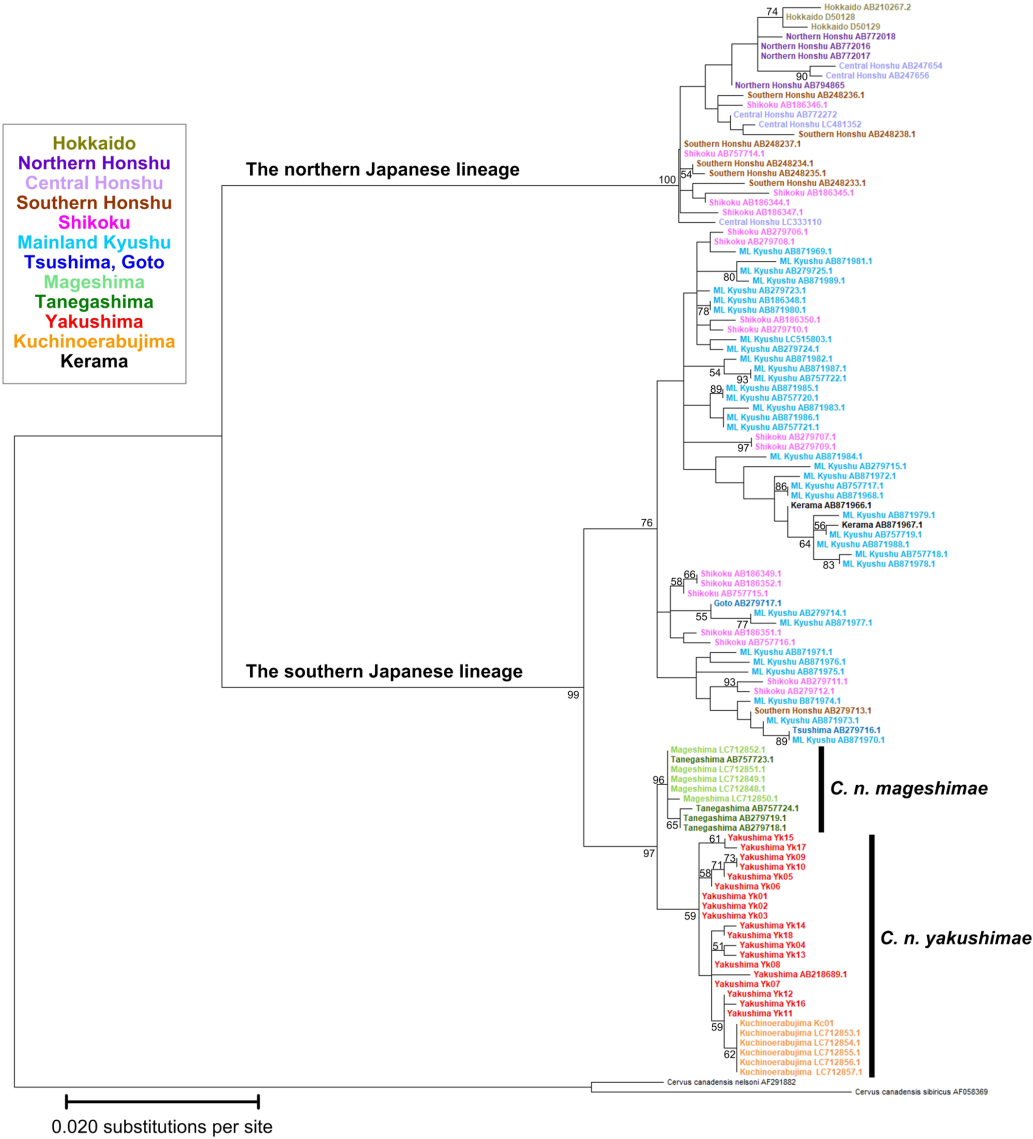
Maximum likelihood tree based on the mitochondrial DNA control region for the 110 haplotypes of the sika deer (*Cervus nippon*) in Japan with Wapiti (*C. canadensis*) as the outgroup. Numbers of nucleotide substitutions per site indicated by scale are the Tamura 3-parameter distances^97^. Numbers on branches indicate bootstrap values after 1,000 replicates. See Supplementary Table S4 for haplotype information.

The molecular phylogenetic tree (Fig. 5) showed that after the sika deer split into the north-south lineage, Yaku sika deer (inhabiting Yakushima and Kuchinoerabujima) and Mage sika deer (inhabiting Tanegashima and Mageshima) diverged from the south lineage and further diverged into the current Yaku sika deer and Mage sika deer.

Based on divergence time estimates of the north–south lineage by Nagata et al.^12^, Kuwayama and Ozawa^19^, and Liu et al.^20^ —0.35 mya, 0.41 mya, and 0.53 mya, respectively—the divergence of this lineage was calibrated at 0.43 mya. Under this calibration, the Yakushima populations diverged from the Honshu population at 0.114 mya, and subsequently, Tanegashima and Yakushima themselves split approximately 37,500 years ago. A simultaneous radiation within the Yakushima population is estimated to have occurred sometime after 4,900–6,500 years ago.

## Discussion

Analysis of a partial control region mtDNA sequence of Yaku sika deer revealed that the haplotype network shows a radial shape centered on Yk01^41^, with most haplotypes connected by a single mutation (Fig. 2a). The mismatch distributions of the whole Yakushima population were unimodal with a peak at three (Fig. 4 left), suggesting recent simultaneous dispersal of subspecies of sika deer on Yakushima^42,43^. The SSD and ruggedness index supported this finding. Of the four categories classified by Grant and Bowen^44^ based on different combinations of small and large haplotype diversity and nucleotide diversity values, populations that showed high haplotype diversity (h > 0.5) and low nucleotide diversity (π < 0.005) indicated that expansion occurred after a sustained period of small population size. The sika deer population in Yakushima met this criterion (Table 1). Moreover, such a demographic history was suggested by the results of the haplotype network and mismatch distribution. However, Tajima’s *D* (*p* = 0.783) and Fu’s *F*s (*p* = 0.338) results did not suggest significant population expansion, contraction, or subdivision (Table 3). These results can be interpreted as the Yaku sika deer population sufficiently recovering from past population reductions and subsequently undergoing further generational changes that reduced the bottleneck and founder effects. In addition, unevenly distributed haplotypes on the island indicate that distinct genetic differentiation occurred among the areas (Table 2, Supplementary Tables S2 and S3 online). Individual Yaku sika deer have a much smaller home range size and are more sedentary than other subspecies of sika deer^33,35^. The presence of predators usually induces prey species to disperse widely and migrate frequently to avoid predation^45–47^; however, this was not observed for the Yaku sika deer in Yakushima, where predators have been absent in the past. Thus, the low genetic exchange between various areas in Yakushima might have induced spatial differences in haplotype structure after population reductions.

SAMOVA revealed two genetic structural populations: northern (Areas B, C, G, and I) and southern (Areas A, D, E, F, and H) (Fig. 3). The boundary of the populations roughly coincides with the thickness of the sediments from the Koya pyroclastic flow, suggesting that the extreme reduction in the Yaku sika deer population (bottleneck) was caused by the Koya pyroclastic flow from the eruption of the Kikai caldera 7,300 years ago^36^. However, the population in Area A did not belong to the northern population, despite Area A being severely affected by the pyroclastic flow. Terada et al.^17^ analyzed microsatellite DNA using a spatially explicit Bayesian model and divided Yaku sika deer into four populations: northern, southern, eastern, and western (which approximately corresponded to Area A). The population in Area A was genetically different from the populations in any other area (Table 2). Therefore, the classification of this population as an independent genetic population is reasonable. People have settled in the few flatlands in low-elevation areas around the steep island of Yakushima. In contrast, permanent settlements have not formed in Area A, which is likely because the characteristics of Area A, including low precipitation^48^, low soil moisture^49^, and particularly low temperatures among the lower elevation areas of the island^50^. Area A is the only lowland designated as a World Heritage Area based on the continuous forest extending from the mountain tops to the coast, which maintains an extremely high deer population density. These conditions may have created a unique habitat that influenced the genetic differentiation of Area A. Genetic diversity in the north was high despite the high impact of the pyroclastic flow; conversely, diversity was relatively low in the south (Table 1). This finding indicates that the ancestral haplotype Yk01 was maintained in a stable subpopulation for a long time in the southern areas, where damage from the pyroclastic flow was low. The SSD and ruggedness index also suggest that the southern population did not experience a bottleneck (Table 3). In contrast, the northern areas were severely damaged by the pyroclastic flow, although very small populations could have survived separately in the intricate mountainous terrain.

Mismatch analysis showed that the northern population had a bimodal distribution with a peak at one and four substitutions (Fig. 4 center). Bimodal mismatch distribution is a common pattern among secondary contacts in fragmented populations^51^. However, given the proximity of the two peaks in the northern population, it is more likely that multiple haplotypes remained after the bottleneck event rather than a possible secondary contact. Indeed, the SSD and raggedness index also indicate that the observed frequency distribution fits the distribution expected under the demographic expansion model. The founder population may have grown faster than the subsequent recovery population^6^. In ungulate species, early recovery of genetic diversity has been observed in founder populations^6,52^. Moreover, genetic diversity in founder populations is higher than that in populations that did not experience recent population reductions^53^. Thus, the small populations in the northern areas (founder populations) may have recovered rapidly with vegetation recovery. Kimura et al.^54^ conducted a radiocarbon analysis of pollen from buried soils at approximately 1,000 m above sea level (asl) in Area H, where the impact of the pyroclastic flow was relatively small, and the results suggested that grassland communities dominated by grasses and ferns were established at some point during the 1,100 years following the pyroclastic flow. Even in the southernmost part of Kyushu (Satsuma and Ohsumi Peninsulas, located approximately 60 km from Yakushima), which was affected by the Koya pyroclastic flow, the previously distributed evergreen broadleaf forests of *Castanopsis* and Lauraceae trees disappeared and did not recover for 600–900 years^55^. Thus, these findings demonstrate that the forest vegetation on Yakushima was almost completely destroyed except in certain south areas of the island and that vast grasslands or sparse forests spread for approximately 1,000 years afterward. With vegetation recovery, phytophagous animal populations should have increased based on the abundant food resources^6,53,56^. The small population that survived in the northern part of the island probably expanded rapidly as the vegetation recovered, and radiated following approximately 6,000 years ago. This finding is almost consistent with the estimated timing of the simultaneous radiation of Yaku sika deer (approximately 4,900–6,500 years ago), which was calculated by calibrating the results of the molecular phylogenetic analysis (Fig. 5) using the divergence date of 0.43 mya between the northern and southern lineages. Yaku sika deer may have acquired high genetic diversity through generational changes and genetic drift sufficient to fix region-specific mutations.

Studies on the strait formation history of these regions suggest that ancient Yakushima—that is, Yakushima prior to its separation from Tanegashima and Magejima—separated from the Paleo-Honshu Island (present-day Honshu, Shikoku, and Mainland Kyushu) 0.1–0.15 mya around MIS5^57,58^. Subsequently, some researchers propose that ancient Yakushima reconnected with Honshu during the Last Glacial Maximum (MIS2)^59,60^, while another posits that the islands remained unconnected following strait formation at 0.1–0.15 mya^57^. Molecular phylogenetic analysis conducted in this study (Fig. 5) suggests that ancient Yakushima was not connected to Honshu during the last glacial period, because of the estimated divergence of Yaku sika deer and Mage sika deer from other southern Japanese sika deer lineages at approximately 0.114 mya.

Although the Yakushima population belong to the southern Japanese lineage in the mtDNA control region, their karyotype is not the same as other populations in the southern lineage (2*n* = 64 or 66 chromosomes) but consistent with that of the northern lineage (2*n* = 68)^21^. In karyotypic polymorphisms of chromosomes in Cervidae, 2*n* = 68 is the basic type^21,61^ and is ancestral to 2*n* = 64 and 2*n* = 66.

The mtDNA control region of sika deer contains repetitive sequences consisting of 38–40 bp called variable number tandem repeated sequences (VNTRs)^12,62,63^. The VNTR copy numbers observed in the sika deer analyzed in this study ranged from three to ten variants, including the original sequence. (Supplementary Table S4 online). The number of VNTR variations in mammals occurs through replication slippages of the original sequence^12,62,64^, with lower repeat copy numbers representing a more ancestral form. Sika deer likely arrived in the Japanese Archipelago from the Eurasian Continent across a land bridge over the East China Sea around 0.43 mya (MIS12)^65^, which is close to the time when sika deer are thought to have split into the north-south lineage^12,19,20^. At that time, both lineages likely had 2*n* = 68 karyotypes and three VNTRs. After ancient Yakushima Islands separated from the Paleo-Honshu Island (present-day Honshu + Shikoku + Mainland Kyushu) at 0.1–0.15 mya^57,58^, a new southern lineage population with 2*n* = 64 and 66 chromosomes may have been originated in the Paleo-Honshu Island (present-day Honshu + Shikoku + Mainland Kyushu). In addition, the old type southern lineage population with 2*n* = 68 chromosomes was likely replaced by the new southern lineage population but remained only on the island of ancient Yakushima.

The origin of the deer inhabiting Kuchinoerabujima is a mystery. Kuchinoerabujima Island is estimated to have formed at approximately 0.58 mya based on dating the oldest tephra found on the island, the Koseda pyroclastic flow, using the glass isothermal plateau fission track method^66,67^. Kuchinoerabujima is surrounded by a sea with a depth of 500–600 m^66^, which indicates that the island has never been connected to any other island or continent since its formation. Of the two haplotypes obtained from deer on Kuchinoerabujima in this study, Kc01 was identical to KUE201–KUE206 (Supplementary Table S4 online) reported by Nagata et al.^68^ (in an 894 bp comparison). Although the read length of Kc02 was rather short (841 bp), the sequence was identical to Yk06 of Yakushima, and the haplotype was detected for the first time on Kuchinoerabujima. Sika deer can swim in the sea and have been reported to cross about 3 km from island to island^69,70^. The distance between Yakushima and Kuchinoerabujima is approximately 12 km. Although the possibility of deer swimming between the islands cannot be excluded, it is unlikely because much more frequent migration should have occurred between Yakushima and Tanegashima. The sea depth between Yakushima and Tanegashima is less than 130 m^57^, and when the sea level dropped during MIS2 (estimated from the bathymetry in Inouchi^57^, the distance between islands likely reached 2.5 km (80 m depth drop) to 5 km (60 m depth drop). However, the haplotype network (Fig. 2) and molecular phylogenetic tree (Fig. 5) clearly distinguish populations of Yaku and Mage sika deer, and common haplotypes have not been found between the two islands. (see also Nagata^25^ and Yamada et al.^14^). Therefore, it is unlikely that sika deer (at least female) migrated between Yakushima and Tanegashima. Calibration of the molecular phylogenetic analysis (Fig. 5) indicated that the Kc01 haplotype, found exclusively on Kuchinoerabujima, was formed approximately 2,100 years ago, during the Yayoi period; therefore, it is early to infer whether they were introduced by humans after Antiquity. Further detailed genetic analyses, including nuclear DNA analyses, are required to address the origin of the deer at Kuchinoerabujima.

In this study, we demonstrated the recovery process of genetic diversity and the reconstruction of local genetic structures after a bottleneck within an island population of deer. Different degrees of past catastrophic impact areas across the island reflected the current local genetic diversity and the mosaic composition of the population structure. These findings suggest these genetically distinct subpopulations—particularly the Area A population—can be treated as independent conservation management units, which is essential for the development of effective long-term monitoring and conservation strategies. The process identified in this study might also have occurred in other populations, thus leading to an early recovery of genetic diversity.

## Materials and methods

### Study sites

Yakushima (30° N, 130° E) is a round island with an area of 504 km^2^ and a circumference of 130 km. It is located 60 km south of Kyushu Island, southern Japan, and lies 18 km west of Tanegashima Island (Fig. 1a,b). Most of the island is steep and mountainous, with Mt. Miyanoura (1,936 m asl) standing at the center of the island and representing the highest mountain in the Kyushu area. The average annual precipitation in Yakushima shows great regional variation, ranging from 2,400 mm in the western coastal region (lowest) to 7,400 mm in the eastern inland region (highest)^48^. The western part of the island presents a continuous vertical distribution of natural vegetation from subtropical evergreen broad-leaved forests in coastal areas to cool-temperate grasslands at the summit, and its unique ecosystem was designated as a World Natural Heritage Site (10,747 ha) in 1993. More than 90% of Yakushima is covered with forest^71^, and most agricultural, residential, and commercial areas are located in low-lying lands at an altitude of less than 150 m asl^72^. Kuchinoerabujima (36 km^2^) is a volcanic island located 12 km west of Yakushima Island. When Mt. Shindake (626 m asl) erupted in 2015, approximately 150 islanders were forced to evacuate from the island, and their return was only possible after seven months. Today, approximately 40% of Yakushima and the entire area of Kuchinoerabujima together form Yakushima National Park.

Between 1.0 and 1.7 mya, Yakushima, along with Tanegashima, was likely connected to Paleo-Honshu Island (present-day Honshu + Shikoku and Mainland Kyushu; Fig. 1a) and the Eurasian Continent^59,73^. According to marine sediment surveys concerning the formation of the strait, an island detached from Honshu—which comprises present-day Shikoku and Kyushu—between 0.15 and 0.1 mya^57,58^, and was subsequently divided into Tanegashima and Yakushima (Fig. 1b). Many remains of the early Jomon period have been found in Yakushima, where human settlements are estimated to have formed at least 7,000 years ago^74^. At approximately 0.58 mya, the volcanic island Kuchinoerabujima was created^66,67^, and was likely never connected to the surrounding islands or the continent (Fig. 1b). The Kikai Caldera, located approximately 30 km northwest of Yakushima (Fig. 1a,b), erupted approximately 7,300 years ago, and a large amount of pyroclastic sediment covered Yakushima and Kuchinoerabujima^36^.

### Sample collection, storage, and DNA extraction

We mainly used deer feces as noninvasive samples for genetic analysis. Fecal samples are the most informative and highly available sources of Yaku sika deer genomic DNA^75^. This approach enabled the collection of genetic material even from steep mountainous regions and wildlife protection areas without relying on conventional invasive methods (e.g., tissue samples from harvested deer). Fresh fecal samples are desirable for obtaining accurate genetic information; however, the large regional differences in deer densities on Yakushima^76,77^ increase the difficulty of collecting fresh feces in low-density areas. However, analyses of brown bear (*Ursus arctos*) feces collected more than one month after defecation^78^ and Asian elephants (*Elephas maximus*) collected eight days after defecation^79^ revealed that the mtDNA levels were sufficient for analysis. Our previous study on Yaku sika deer showed that feces collected within three days after defecation can be used to analyze nuclear microsatellite DNA regardless of temperature or rainfall^80^. Because mtDNA has overwhelmingly more copies per cell than nuclear DNA, it generally retains its analytical efficiency longer than nuclear microsatellite DNA^81^. Thus, feces collected several days after defecation was acceptable for our analysis.

Genomic DNA was collected from Yaku sika deer from February 2004 to April 2017. We collected 373 fecal samples—in addition to 32 hair samples and 14 tissue fragments—from Yakushima. We also collected five fecal samples from Kuchinoerabujima in July 2004. In the western lowlands of Yakushima, we performed deer tracking and collected fresh feces immediately after defecation because deer in this area are habituated to humans and are not afraid of researchers; therefore, direct observations are relatively easy^31,32^. In other areas, we walked through the forest to search for fecal remains on the ground and collected only feces that appeared to be relatively fresh based on their appearance, as suggested by Agetsuma-Yanagihara et al.^80^. In addition, we used hairs collected from deer captured for transmitter installation^31,32,35^, fallen hair bundles in the forest, and tissue fragments collected from carcasses found in the forest and from individuals culled as pests by the local government. Deer capture and handling protocols for the transmitter installation had been reviewed and permitted annually by the Ministry of the Environment, Government of Japan and the Kagoshima Prefectural Government throughout the study period.

Feces collected from February 2004 to May 2013 were air-dried at 15 °C to 25 °C and stored at −20 °C to −30 °C. The surface layer of the frozen feces was scraped with a sterile scalpel on ice, and DNA was extracted and purified using the QIAamp DNA Stool Mini Kit (QIAGEN, Hilden, Germany). From May 2015 to April 2017, the surface of feces, which is thought to contain adhered intestinal cells^82^, was rubbed several times with a sterile cotton swab, which was then rinsed in a 2 mL tube containing conditioned lysis buffer^83^. The solution was stored at room temperature in a cool, dark place^80^. DNA was extracted and purified from the fecal solutions using the QIAamp DNA Stool Mini Kit (QIAGEN) or the QIAamp Fast DNA Stool Mini Kit (QIAGEN). Hair samples were washed thoroughly with ethanol (> 70%) and stored at −20 °C to −30 °C, and DNA was extracted and purified using ISOHAIR (Nippon Gene Co., Ltd., Tokyo, Japan). Immersed tissue fragments were stored in ethanol (> 70%) at −20 °C to −30 °C, and DNA was extracted and purified using a DNeasy Blood & Tissue Kit (QIAGEN). The purified DNA was stored at −20 °C to −30 °C if not analyzed immediately.

### PCR direct sequencing of the mtDNA control region

The control region of mtDNA was amplified using the primer pair LD5 (5′-AAGCCATAGCCCCACTATCAA-3′) and H597 (5′-AGGCATTTTCAGTGCCTTGCTTTG-3′)^84^ and the Tks Gflex DNA Polymerase PCR enzyme (Takara Bio Inc., Shiga, Japan), which is an alpha-type enzyme with both proofreading and polymerase activity^85,86^. In addition, previous study revealed that Tks Gflex has high fidelity^87^. PCR amplifications were carried out in a total volume of 20 µL, which contained 2 µL template DNA, 0.4 µL Tks Gflex DNA Polymerase, and 0.05 µL of each 100 µM primer (LD5 and H597). PCR was performed on a thermal cycler (TaKaRa PCR Thermal Cycler Dice Touch; Takara Bio Inc.). The following PCR protocol was used: after initial incubation at 94 °C for 1 min, 45 amplification cycles were performed with denaturation at 98 °C for 10 s, annealing at 55 °C for 15 s, and extension at 68 °C for 40 s. Successful PCR amplification was confirmed by electrophoresis of PCR products on a 1.5% agarose gel containing GelGreen (Biotium, Fremont, CA, USA), followed by visualization under UV illumination.

PCR products were purified with the High Pure PCR Product Purification Kit (Roche Diagnostics K.K., Tokyo, Japan) and sequenced using the BigDye Terminator v3.1 Cycle Sequencing Kit (Applied Biosystems, Waltham, MA, USA). Cycle sequencing reactions were carried out in a total volume of 10 µL, which contained 2 µL PCR product, 1 µL Ready Reaction Mix, 2 µL Sequencing Buffer, and 1 µL primer (1 µM; LD5 or H597). After initial incubation at 96 °C for 1 min, 50 amplification cycles were performed with denaturation at 96 °C for 10 s, annealing at 50 °C for 5 s, and extension at 60 °C for 2 min. DNA fragments were sequenced using an ABI3130 Genetic Analyzer (Applied Biosystems).

### Data analysis

We visually confirmed the sequencing chromatograms using MEGAX v10.1.6^88^ and determined the nucleotide sequence of each sample. Haplotypes for each sample were determined using DnaSP ver. 6.12.03^89^. Fecal samples collected within a 200 m radius that exhibited completely identical sequences and could not be determined to originate from different individuals based on external appearance, such as size and shape, were excluded from subsequent genetic analyses. For the haplotypes obtained in this study (18 from Yakushima and 1 from Kuchinoerabujima excluding Kc02) and those found in the sequence data of sika deer in Japan registered in the NCBI (Supplementary Table S4 online), we reconstructed median-joining networks^90^ using Network 10.2.0.0 (https://www.fluxus-engineering.com/sharenet.htm). The positional definition and counting method of VNTRs differ among studies by Randi et al.^63^, Nagata et al.^12^, and Cook et al.^62^; in the present study, we followed the sites and method of Randi et al.^63^. A total of 42 distinct VNTR units were identified from all sequence data used in the network analysis, with repeat copy numbers (including the original) ranging from three to ten. The original VNTR sequence and the two copies are commonly found across all subspecies of sika deer, as well as in the Manchurian and North American subspecies of elk (*C. canadensis*), rusa deer (*C. timorensis*), and sambar deer (*C. unicolor*)^63^. In contrast, the presence of three or more copies is a unique feature observed only in sika deer populations in the Japanese Archipelago^12,63^. Thus, the original and the two-copy sequences represent ancestral forms, and subsequent copies are considered to be derived variants. Accordingly, we used only the original VNTR sequence and its two copies in the haplotype network and molecular phylogenetic analyses in this study. The sites where the bases could not be deciphered were excluded from the analysis, and the alignment gap was treated as a fifth base.

To examine the genetic subpopulation structure of Yaku sika deer in Yakushima, we divided Yakushima into nine areas from A to I (Fig. 1b) based on the following habitat conditions: impact of the pyroclastic flow caused by the giant eruption of the Kikai Caldera approximately 7,300 years ago^36^; forest logging history over the past 100 years^91^; distribution of annual precipitation^48^; altitude; and status as a World Heritage Area (strictest natural protection area). We also searched for genetic boundaries in the aforementioned nine areas based on SAMOVA ver. 2.0^92^. To examine the relationship between the results of SAMOVA and impact of the Koya pyroclastic flow, the distribution of the sediment thickness over Yakushima was estimated by spatially interpolating (Gaussian model, 5 km mesh) the thickness measured by Geshi^36^ using ArcGIS ver. 9.3 (ESRI Inc.).

We employed Arlequin v3.5.2^93^ to calculate the haplotype frequency, haplotype diversity (*h*), nucleotide diversity (*π*), and pairwise *F*_ST_ values for each area and performed AMOVA. We computed Tajima’s *D*^94^ and Fu’s *F*s^95^ values to summarize the haplotype frequency, and their randomness and neutrality were tested using Arlequin v3.5.2. Mismatch analysis^42,43^ was performed using Arlequin v3.5.2^93^ to examine how each sample population formed the current population. The SSD between the observed and expected distributions and ruggedness index (*r*)^96^ were used to assess the goodness of fit of the sudden expansion model in the mismatch analysis. Moreover, we used MEGA X v10.1.6^88^ to construct phylogenetic trees of sika deer in Japan (Supplementary Table S4 online) with Wapiti (*C. canadensis*) as the outgroup. The best nucleotide substitution model for phylogenetic tree construction was estimated using the maximum likelihood method, which was complemented by MEGA X v10.1.6^88^.

## Supplementary Information

Below is the link to the electronic supplementary material.

Supplementary Material 1

Supplementary Material 2

## Data Availability

All data analyzed during this study are included in this published article and its supplementary information files.

## References

[1] Bouzat, J. L. et al. Genetic evaluation of a demographic bottleneck in the greater prairie chicken. *Conserv. Biol.* **12**, 836–843. 10.1111/j.1523-1739.1998.97164.x (1998).

[2] Saccheri, I. et al. Inbreeding and extinction in a butterfly metapopulation. *Nature* **392**, 491–494. 10.1038/33136 (1998).

[3] Spielman, D., Brook, B. W. & Frankham, R. Most species are not driven to extinction before genetic factors impact them. *Proc. Natl. Acad. Sci.* **101**, 15261–15264. 10.1073/pnas.0403809101 (2004). 10.1073/pnas.0403809101 PMC524053 15477597

[4] Booy, G., Hendriks, R. J. J., Smulders, M. J. M., Groenendael, J. M. & Vosman, B. Genetic diversity and the survival of populations. *Plant Biol.* **2**, 379–395. 10.1055/s-2000-5958 (2000).

[5] Pang, J., Hoelzel, A. R., Song, Y., Zeng, Z. & Zhang, Y. Lack of mtDNA control region variation in Hainan Eld’s deer: Consequence of a recent population bottleneck? *Conserv. Genet.* **4**, 109–112. 10.1023/A:1021817925740 (2003).

[6] Lovatt, F. M. & Hoelzel, A. R. Impact on reindeer (*Rangifer tarandus*) genetic diversity from two parallel population bottlenecks founded from a common source. *Evol. Biol.* **41**, 240–250. 10.1007/s11692-013-9263-2 (2014).

[7] Harris, R. B. *Cervus nippon: the IUCN red list of threatened species 2015*. e.T41788A22155877. 10.2305/IUCN.UK.2015-2.RLTS.T41788A22155877.en (2015).

[8] Agetsuma, N. Are deer populations increasing abnormally? *Biol. Sci.* **65**, 108–116 (2013). (in Japanese).

[9] Agetsuma, N. A simple method for calculating minimum estimates of previous population sizes of wildlife from hunting records. *PLoS One*. **13**, e0198794. 10.1371/journal.pone.0198794 (2018). 29894510 10.1371/journal.pone.0198794 PMC5997341

[10] Oka, T. et al. The process of population expansion of sika deer. In *Sika Deer: Life History Plasticity and Management* (eds Kaji, K., Uno, H. & Iijima, H.) 11–23. 10.1007/978-981-16-9554-4_2 (Springer Nature, 2022).

[11] Ohtaishi, N. Preliminary memorandum of classification, distribution and geographic variation on Sika deer. *Mammalian Sci.* **26**, 13–17. 10.11238/mammalianscience.26.2_13 (1986). (in Japanese).

[12] Nagata, J. et al. Two genetically distinct lineages of the sika deer, *Cervus nippon*, in Japanese islands: comparison of mitochondrial D-loop region sequences. *Mol. Phylogenet Evol.* **13**, 511–519. 10.1006/mpev.1999.0668 (1999). 10620409 10.1006/mpev.1999.0668

[13] Yamada, M. et al. Distribution of two distinct lineages of sika deer (*Cervus nippon*) on Shikoku Island revealed by mitochondrial DNA analysis. *Mammal Study*. **31**, 23–28. 10.3106/1348-6160(2006)31[23:DOTDLO]2.0.CO;2 (2006).

[14] Yamada, M., Hosoi, E., Nagata, J., Tamate, H. B. & Tado, H. Phylogenetic relationship of the southern Japan lineages of the sika deer (*Cervus nippon*) in Shikoku and Kyushu Islands, Japan. *Mammal Study*. **32**, 121–127. 10.3106/1348-6160(2007)32[121:PROTSJ]2.0.CO;2 (2007).

[15] Goodman, S. J. et al. Bottlenecks, drift and differentiation: the population structure and demographic history of sika deer (*Cervus nippon*) in the Japanese archipelago. *Mol. Ecol.* **10**, 1357–1370. 10.1046/j.1365-294X.2001.01277.x (2001). 11412360 10.1046/j.1365-294x.2001.01277.x

[16] Hata, S. et al. Detection of genetic segregation in sika deer (*Cervus nippon*) by tandem repeat variations in the mitochondrial DNA D-loop region. *J. For. Res.* **24**, 325–329. 10.1080/13416979.2019.1662877 (2019).

[17] Terada, C., Yahara, T., Kuroiwa, A. & Saitoh, T. Spatial genetic structure of the sika deer (*Cervus nippon*) population on Yakushima: Significant genetic differentiation on a small island. *Mammal Study*. **46**, 225–235. 10.3106/ms2020-0088 (2021).

[18] Takagi, T. et al. Development of paternally-inherited Y chromosome simple sequence repeats of sika deer and their application in genetic structure, artificial introduction, and interspecific hybridization analyses. *Popul. Ecol.* **64**, 150–160. 10.1002/1438-390X.12109 (2022).

[19] Kuwayama, R. & Ozawa, T. Phylogenetic relationships among european red deer, wapiti, and sika deer inferred from mitochondrial DNA sequences. *Mol. Phylogenet. Evol.* **15**, 115–123. 10.1006/mpev.1999.0731 (2000). 10764539 10.1006/mpev.1999.0731

[20] Liu, H. et al. Population genomics of sika deer reveals recent speciation and genetic selective signatures during evolution and domestication. *BMC Genom.* **26**, 364. 10.1186/s12864-025-11541-w (2025). 10.1186/s12864-025-11541-w PMC11987376 40217144

[21] Harada, M. Caused by the emergence of Robertsonian rearrangements in intersubspecies hybrid. Report on research results of grants-in-aid for scientific research (in Japanese with English summary) (2010).

[22] Omura, Y., Fukumoto, Y. & Ohtaki, K. Chromosome polymorphism in Japanese sika, *Cervus* (*Sika*) *nippon*. *Jpn. J. Vet. Sci.* **45**, 23–30. 10.1292/jvms1939.45.23 (1983). 10.1292/jvms1939.45.23 6865171

[23] Fukui, E. & Yoshizawa, M. Some aspects concerning cytogenetics and molecular genetics in native Sika deer. *J. Anim. Genet.* **36**, 117–121. 10.5924/abgri2000.36.117 (2008). (in Japanese)

[24] Whitehead, G. K. *The Whitehead Encyclopedia of Deer* (Swan Hill, 1993).

[25] Nagata, J. Two genetically distinct lineages of the Japanese sika deer based on mitochondrial control regions. In *Sika Deer: Biology and Management of Native and Introduced Populations* (eds McCullough, D. R., Takatsuki, S. & Kaji, K.) 27–41. 10.1007/978-4-431-09429-6_3 (Springer Japan, 2009).

[26] Terada, C. & Saitoh, T. Phenotypic and genetic divergence among island populations of sika deer (*Cervus nippon*) in southern Japan: a test of the local adaptation hypothesis. *Popul. Ecol.* **60**, 211–221. 10.1007/s10144-018-0607-8 (2018).

[27] Terada, C. Geographic variations of morphological and genetic features among island populations of the sika deer (*Cervus nippon*) in southern Japan: ecological and historical nonexchangeability. PhD thesis, Hokkaido Univ. (2012).

[28] Terada, C., Tatsuzawa, S. & Saitoh, T. Ecological correlates and determinants in the geographical variation of deer morphology. *Oecologia* **169**, 981–994. 10.1007/s00442-012-2270-7 (2012). 22327615 10.1007/s00442-012-2270-7

[29] Nature Conservation Bureau Environment Agency, Japan. *Conservation Reports of the Yaku-shima Wilderness Area, Kyushu, Japan.* (in Japanese) Tokyo. 10.11501/3948418 (1984).

[30] Kubo, M. O. Trends in morphological evolution of insular artiodactyls. *Biol. Sci.* **66**, 30–41 (2014). (in Japanese).

[31] Agetsuma, N., Agetsuma-Yanagihara, Y. & Takafumi, H. Food habits of Japanese deer in an evergreen forest: Litter-feeding deer. *Mamm. Biol*. **76**, 201–207. 10.1016/j.mambio.2010.04.002 (2011).

[32] Agetsuma, N., Agetsuma-Yanagihara, Y., Takafumi, H. & Nakaji, T. Plant constituents affecting food selection by sika deer. *J. Wildl. Manag.* **83**, 669–678. 10.1002/jwmg.21615 (2019).

[33] Agetsuma, N., Agetsuma-Yanagihara, Y. & Sugiura, H. Increase and decline in the density index of Japanese sika deer (*Cervus nippon*) over 18 years in an evergreen broad-leaved forest with no hunting pressure in the Natural World Heritage Area of Yakushima, Japan. *Jpn. J. Conserv. Ecol.* **26**, 87–100. 10.18960/hozen.1923 (2021). (in Japanese with English abstract).

[34] Hayashi, S. et al. Variation and process of life history evolution in insular dwarfism as revealed by a natural experiment. *Front. Earth Sci.* **11**, 1–15. 10.3389/feart.2023.1095903 (2023).

[35] Agetsuma, N., Agetsuma-Yanagihara, Y. & Takafumi, H. Ranging patterns of Japanese sika deer in a warm temperate forest of Yakushima. In *Annual Report of Sustainability and Biodiversity Assessment on Forest Utilization Options* (eds. Research Institute for Humanity and Nature) 4–5 (Research Institute for Humanity and Nature, 2005) (in Japanese).

[36] Geshi, N. Distribution and flow mechanisms of the 7.3 ka Koya pyroclastic flow deposits covering Yakushima Island, Kagoshima Prefecture. *J. Geogr.* **118**, 1254–1260. 10.5026/jgeography.118.1254 (2009). (in Japanese with English abstract).

[37] Hayaishi, S. & Kawamoto, Y. Low genetic diversity and biased distribution of mitochondrial DNA haplotypes in the Japanese macaque (*Macaca fuscata yakui*) on Yakushima Island. *Primates* **47**, 158–164. 10.1007/s10329-005-0169-1 (2006). 16418879 10.1007/s10329-005-0169-1

[38] Vawter, L. & Brown, W. M. Nuclear and mitochondrial DNA comparisons reveal extreme rate variation in the molecular clock. *Science* **234**, 194–196. 10.1126/science.3018931 (1986). 3018931 10.1126/science.3018931

[39] Moritz, C. Applications of mitochondrial DNA analysis in conservation: a critical review. *Mol. Ecol.* **3**, 401–411. 10.1111/j.1365-294X.1994.tb00080.x (1994).

[40] Wada, K., Nishibori, M. & Yokohama, M. The complete nucleotide sequence of mitochondrial genome in the Japanese Sika deer (*Cervus nippon*), and a phylogenetic analysis between Cervidae and Bovidae. *Small Ruminant Res.* **69**, 46–54. 10.1016/j.smallrumres.2005.12.002 (2007).

[41] Slatkin, M. & Hudson, R. R. Pairwise comparisons of mitochondrial DNA sequences in stable and exponentially growing populations. *Genetics* **129**, 555–562. 10.1093/genetics/129.2.555 (1991). 1743491 10.1093/genetics/129.2.555 PMC1204643

[42] Rogers, A. R. & Harpending, H. Population growth makes waves in the distribution of pairwise genetic differences. *Mol. Biol. Evol.* **9**, 552–569. 10.1093/oxfordjournals.molbev.a040727 (1992). 1316531 10.1093/oxfordjournals.molbev.a040727

[43] Rogers, A. R., Fraley, A. E., Bamshad, M. J., Watkins, W. S. & Jorde, L. B. Mitochondrial mismatch analysis is insensitive to the mutational process. *Mol. Biol. Evol.* **13**, 895–902. 10.1093/molbev/13.7.895 (1996). 8751998 10.1093/molbev/13.7.895

[44] Grant, W. S. & Bowen, B. W. Shallow population histories in deep evolutionary lineages of marine fishes: insights from sardines and anchovies and lessons for conservation. *J. Hered.* **89**, 415–426. 10.1093/jhered/89.5.415 (1998).

[45] Edwards, J. Diet shifts in moose due to predator avoidance. *Oecologia* **60**, 185–189. 10.1007/BF00379520 (1983). 28310485 10.1007/BF00379520

[46] Fryxell, J. M. & Sinclair, A. R. E. Causes and consequences of migration by large herbivores. *Trends Ecol. Evol.* **3**, 237–241. 10.1016/0169-5347(88)90166-8 (1988). 21227239 10.1016/0169-5347(88)90166-8

[47] Mao, J. S. et al. Habitat selection by elk before and after wolf reintroduction in Yellowstone National Park. *J. Wildl. Manag.* **69**, 1691–1707. 10.2193/0022-541X(2005)69[1691:HSBEBA]2.0.CO;2 (2005).

[48] Takahara, H. & Matsumoto, J. Climatological study of precipitation distribution in Yaku-shima Island, southern Japan. *J. Geogr. (Chigaku Zasshi)*. **111**, 726–746. 10.5026/jgeography.111.5_726 (2002). (in Japanese with English abstract)

[49] Aiba, S. Structure and function of Yakushima forests. In *World Heritage Yakushima: Subtropical Nature and Ecosystem* (eds. Osawa, M., Tagawa, H., & Yamagiwa, J.) 102–117 (Asakura Publishing Co., Ltd, 2006) (in Japanese).

[50] Kurihara, Y. & Aiba, S. Data and supplementary information for "Meteorological data for Nagata, Yakushima Island, Japan, during 2005–2014". *figshare* (2020). 10.6084/M9.FIGSHARE.11662368 (in Japanese with English abstract)

[51] Avise, J. C. *Phylogeography: the History and Formation of Species* (Harvard University Press, 2000).

[52] Deyoung, R. W. et al. Genetic consequences of white-tailed deer (*Odocoileus virginianus*) restoration in Mississippi. *Mol. Ecol.* **12**, 3237–3252. 10.1046/j.1365-294X.2003.01996.x (2003). 14629342 10.1046/j.1365-294x.2003.01996.x

[53] Broders, H. G., Mahoney, S. P., Montevecchi, W. A. & Davidson, W. S. Population genetic structure and the effect of founder events on the genetic variability of moose, *Alces alces*, in Canada. *Mol. Ecol.* **8**, 1309–1315. 10.1046/j.1365-294X.1999.00695.x (1999). 10447871 10.1046/j.1365-294x.1999.00695.x

[54] Kimura, K., Ooi, N. & Suzuki, S. Evidence of vegetation recovery on Yakushima Island after the major holocene eruption at the Kikai Caldera, as revealed by the pollen record of buried soils under the old-growth *Cryptomeria Japonica* forest. *Jpn. Assoc. Hist. Bot.* **4**, 13–23. 10.34596/hisbot.4.1_13 (1996).

[55] Sugiyama, S. The impact of the Kikai-Akahoya explosive eruption on vegetation in southern Kyushu, Japan, clarified by phytolith studies. *Quat. Res. (Daiyonki-kenkyu)*. **41**, 311–316. 10.4116/jaqua.41.311 (2002). (in Japanese with English abstract).

[56] Mays, H. L. et al. Genomic analysis of demographic history and ecological niche modeling in the endangered Sumatran rhinoceros *Dicerorhinus sumatrensis*. *Curr. Biol.* **28**, 70–76. 10.1016/j.cub.2017.11.021 (2018). 29249659 10.1016/j.cub.2017.11.021 PMC5894340

[57] Inouchi, Y. Sediments and Qµaternary sedimentological history of the Osumi Strait and its vicinity, in relation to the evolution of the Osumi Strait. *Bull. Geol. Surv. Japan*. **32**, 693–716. https://www.gsj.jp/data/bull-gsj/32-12_01.pdf (1981). (in Japanese with English abstract)

[58] Ohshima, K. The history of straits around the Japanese Islands in the late-Quaternary. *Quat. Res. (Daiyonki-Kenkyu)*. **29**, 193–208. 10.4116/jaqua.29.193 (1990). (in Japanese with English abstract)

[59] Kizaki, K. & Oshiro, I. Paleogeography of the Ryukyu Islands. *Mar. Sci.* **9**, 542–549 (1977). (in Japanese with English abstract).

[60] Ota, H. Geographic patterns of endemism and speciation in amphibians and reptiles of the Ryukyu Archipelago, Japan, with special reference to their paleogeographical implications. *Popul. Ecol.* **40**, 189–204. 10.1007/BF02763404 (1998).

[61] Fontana, F. & Rubini, M. Chromosomal evolution in Cervidae. *Biosystems* **24**, 157–174. 10.1016/0303-2647(90)90008-O (1990). 2249009 10.1016/0303-2647(90)90008-o

[62] Cook, C. E., Wang, Y. & Sensabaugh, G. A mitochondrial control region and cytochrome *b* phylogeny of sika deer (*Cervus nippon*) and report of tandem repeats in the control region. *Mol. Phylogenet. Evol.* **12**, 47–56. 10.1006/mpev.1998.0593 (1999). 10222160 10.1006/mpev.1998.0593

[63] Randi, E., Mucci, N., Claro-Hergueta, F., Bonnet, A. & Douzery, E. J. P. A mitochondrial DNA control region phylogeny of the Cervinae: speciation in *Cervus* and implications for conservation. *Anim. Conserv.* **4**, 1–11. 10.1017/S1367943001001019 (2001).

[64] Gemmell, N. J., Western, P. S., Watson, J. M. & Graves, J. A. Evolution of the mammalian mitochondrial control region–comparisons of control region sequences between monotreme and therian mammals. *Mol. Biol. Evol.* **13**, 798–808. 10.1093/oxfordjournals.molbev.a025640 (1996). 8754216 10.1093/oxfordjournals.molbev.a025640

[65] Kawamura, Y. Fossil record of sika deer in Japan. In *Sika Deer: Biology and Management of Native and Introduced Populations* (eds McCullough, D. R., Takatsuki, S. & Kaji, K.) 11–25. 10.1007/978-4-431-09429-6_2 (Springer Japan, 2009).

[66] Geshi, N. & Kobayashi, T. Geological Survey of Japan, AIST, Tsukuba. *Geological Map of Kuchinoerabujima Volcano; Geological Map of Volcano 14*. https://www.gsj.jp/data/VOLC/PDF/GSJ_MAP_VOLC_14_2007_D.pdf (2007). (in Japanese with English summary).

[67] Moriwaki, H., Westgate, J. A., Sandhu, A. S., Preece, S. J. & Arai, F. New glass fission-track ages of Middle Pleistocene tephras on Yakushima Island, southern Japan. *Quat. Int.* **178**, 128–137. 10.1016/j.quaint.2006.11.013 (2008).

[68] Nagata, J., Watari, Y., Takagi, T., Tatsuzawa, S. & Kaneko, S. Genetic identification of the origin of introduced deer on Kikai-jima Island. *Mamm. Sci.* **63**, 109–117. 10.11238/mammalianscience.63.109 (2023). (in Japanese with English abstract).

[69] Yamashiro, A., Endo, A., Kuwataka, H., Matsumoto, Y. & Yamashiro, T. Geographic origin and genetic structure of introduced sika deer, Kerama deer (*Cervus nippon keramae*) on Ryukyus inferred from mitochondrial DNA sequences. *Mammal Study*. **40**, 187–192. 10.3106/041.040.0306 (2015).

[70] Takagi, T., Matsumoto, Y., Koda, R. & Tamate, H. B. Bi-directional movement of deer between Tomogashima islands and the western part of the Kii Peninsula, Japan, with special reference to hybridization between the Japanese sika deer (*Cervus nippon centralis*) and the introduced exotic deer. *Mammal Study*. **45**, 133–141. 10.3106/ms2019-0048 (2020).

[71] Yakushima Town. *Statistical Reports of Yakushima Town in 2020 *(in Japanese) (Yakushima Town, 2021).

[72] Geospatial Information Authority of Japan. 1:200,000 land use map. (1985).

[73] Kimura, M. Quaternary paleogeography of the Ryukyu Arc. *J. Geogr.* **105**, 259–285. 10.5026/jgeography.105.3_259 (1996). (in Japanese with English abstract).

[74] Yaku Town Local History Compilation Committee. *Local Chronicle of Yaku Town, Volume 1, Local Chronicle of Rural Community *(in Japanese) (Yaku Town board of education, 1993).

[75] Hanya, G. et al. Morphometric and genetic determination of age class and sex for fecal pellets of sika deer (*Cervus nippon*). *Mammal Study*. **42**, 1–8. 10.3106/041.042.0406 (2017).

[76] Agetsuma, N., Koda, R., Tsujino, R. & Agetsuma-Yanagihara, Y. Impact of anthropogenic disturbance on the density and activity pattern of deer evaluated with respect to spatial scale-dependency. *Mamm. Biol*. **81**, 130–137. 10.1016/j.mambio.2015.09.005 (2016).

[77] Koda, R., Agetsuma, N., Agetsuma-Yanagihara, Y., Tsujino, R. & Fujita, N. A proposal of the method of deer density estimate without fecal decomposition rate: a case study of fecal accumulation rate technique in Japan. *Ecol. Res.* **26**, 227–231. 10.1007/s11284-010-0757-4 (2011).

[78] Murphy, M. A., Kendall, K. C., Robinson, A. & Waits, L. P. The impact of time and field conditions on brown bear (*Ursus arctos*) faecal DNA amplification. *Conserv. Genet.* **8**, 1219–1224. 10.1007/s10592-006-9264-0 (2007).

[79] Fernando, P., Pfrender, M. E., Encalada, S. E. & Lande, R. Mitochondrial DNA variation, phylogeography and population structure of the Asian elephant. *Heredity*. **84**, 362–372. 10.1046/j.1365-2540.2000.00674.x (2000). 10762406 10.1046/j.1365-2540.2000.00674.x

[80] Agetsuma-Yanagihara, Y., Inoue, E. & Agetsuma, N. Effects of time and environmental conditions on the quality of DNA extracted from fecal samples for genotyping of wild deer in a warm temperate broad-leaved forest. *Mammal Res.* **62**, 201–207. 10.1007/s13364-016-0305-x (2017).

[81] Inoue, E. DNA analysis using noninvasive samples: Methods of sample collection, DNA extraction, PCR amplification and kinship analysis. *Primate Res.* **31**, 3–18. 10.2354/psj.31.007 (2015). (in Japanese with English summary).

[82] Takenaka, O. DNA polymorphisms reveal kin relationships among chimpanzees. *Primate Res.* **9**, 125–134. 10.2354/psj.9.2_125 (1993). (in Japanese with English summary).

[83] White, P. S. & Densmore, L. D. Mitochondrial DNA isolation. In *Molecular Genetic Analysis of Populations: A Practical Approach* (ed Hoezel, A. R.) 29–58 (Oxford University Press, 1992).

[84] Nagata, J., Masuda, R., Kaji, K., Kaneko, M. & Yoshida, M. C. Genetic variation and population structure of the Japanese sika deer (*Cervus nippon*) in Hokkaido Island, based on mitochondrial D-loop sequences. *Mol. Ecol.* **7**, 871–877. 10.1046/j.1365-294x.1998.00404.x (1998). 9691488 10.1046/j.1365-294x.1998.00404.x

[85] Ishige, T. et al. Evaluation of analytical factors associated with targeted *MEFV* gene sequencing using long-range PCR/massively parallel sequencing of whole blood DNA for molecular diagnosis of Familial Mediterranean fever. *Clin. Chim. Acta*. **495**, 562–569. 10.1016/j.cca.2019.06.001 (2019). 31173732 10.1016/j.cca.2019.06.001

[86] Shen, Z. C., Chen, L., Chen, L. & Li, Y. X. Information from the mitochondrial genomes of two egg parasitoids, *Gonatocerus* sp. and *Telenomus* sp., reveals a controversial phylogenetic relationship between Mymaridae and Scelionidae. *Genomics*. **111**, 1059–1065. 10.1016/j.ygeno.2018.06.009 (2019). 31533898 10.1016/j.ygeno.2018.06.009

[87] Kubo, H., Otsuka, M. & Kadokawa, H. Sexual polymorphisms of vomeronasal 1 receptor family gene expression in bulls, steers, and estrous and early luteal-phase heifers. *J. Vet. Med. Sci.* **78**, 271–279. 10.1292/jvms.15-0300 (2016). 26477467 10.1292/jvms.15-0300 PMC4785117

[88] Kumar, S., Stecher, G., Li, M., Knyaz, C. & Tamura, K. MEGA X: Molecular Evolutionary Genetics Analysis across computing platforms. *Mol. Biol. Evol.* **35**, 1547–1549. 10.1093/molbev/msy096 (2018). 29722887 10.1093/molbev/msy096 PMC5967553

[89] Rozas, J. et al. DnaSP 6: DNA sequence polymorphism analysis of large data sets. *Mol. Biol. Evol.* **34**, 3299–3302. https://doi.org/10.1093/molbev/msx248 (2017). 29029172 10.1093/molbev/msx248

[90] Bandelt, H. J., Forster, P. & Rohl, A. Median-joining networks for inferring intraspecific phylogenies. *Mol. Biol. Evol.* **16**, 37–48. 10.1093/oxfordjournals.molbev.a026036 (1999). 10331250 10.1093/oxfordjournals.molbev.a026036

[91] Miura, K. Nature conservation of Yakushima. *Monkey*. **28**, 64–69 (1984). (in Japanese).

[92] Dupanloup, I., Schneider, S. & Excoffier, L. A simulated annealing approach to define the genetic structure of populations. *Mol. Ecol.* **11**, 2571–2581. 10.1046/j.1365-294X.2002.01650.x (2002). 12453240 10.1046/j.1365-294x.2002.01650.x

[93] Excoffier, L. & Lischer, H. E. L. Arlequin suite ver 3.5: a new series of programs to perform population genetics analyses under Linux and Windows. *Mol. Ecol. Resour.* **10**, 564–567. 10.1111/j.1755-0998.2010.02847.x (2010). 21565059 10.1111/j.1755-0998.2010.02847.x

[94] Tajima, F. Statistical method for testing the neutral mutation hypothesis by DNA polymorphism. *Genetics* **123**, 585–595. 10.1093/genetics/123.3.585 (1989). 2513255 10.1093/genetics/123.3.585 PMC1203831

[95] Fu, Y. X. Statistical tests of neutrality of mutations against population growth, hitchhiking and background selection. *Genetics* **147**, 915–925. 10.1093/genetics/147.2.915 (1997). 9335623 10.1093/genetics/147.2.915 PMC1208208

[96] Harpending, H. C. Signature of ancient population growth in a low-resolution mitochondrial DNA mismatch distribution. *Hum. Biol.* **66**, 591–600 (1994). 8088750

[97] Tamura, K. Estimation of the number of nucleotide substitutions when there are strong transition-transversion and G + C-content biases. *Mol. Biol. Evol.* **9**, 678–687. 10.1093/oxfordjournals.molbev.a040752 (1992). 1630306 10.1093/oxfordjournals.molbev.a040752

[98] Satoh, S. S. et al. Origin of sika deer (*Cervus nippon*) observed in Yamagata Prefecture. *Mamm. Sci.* **53**, 131–137. 10.11238/mammalianscience.53.131 (2013). (in Japanese with English abstract).

[99] Yoshio, M. et al. Spatially heterogeneous distribution of mtDNA haplotypes in a sika deer (*Cervus nippon*) population on the Boso Peninsula, central Japan. *Mammal Study*. **33**, 59–69. 10.3106/1348-6160(2008)33[59:SHDOMH]2.0.CO;2 (2008). .

[100] Yamazaki, Y. Genetic population structure of sika deer, *Cervus nippon*, derived from multiple origins, around Toyama Prefecture of Japan. *Zool. Sci.* **35**, 215–221. 10.2108/zs170187 (2018). 10.2108/zs170187 29882493

[101] Nagata, J., Yasuda, M. & Yamashiro, A. Genetic analysis of a newly established deer population expanding in the Sasebo area in Nagasaki Prefecture, Japan reveals no evidence of genetic disturbance by Formosan sika deer. *Mammal Study*. **46**, 251–263. 10.3106/ms2020-0084 (2021).

