# Supplementary material for: Post-bottleneck increase in mitochondrial DNA diversity in Yaku sika deer (Cervus nippon yakushimae) on Yakushima Island, Japan
Source: Sci Rep. 2025 Nov 12;15:39630. doi: 10.1038/s41598-025-23191-9 (PMC12612164; doi:10.1038/s41598-025-23191-9)
Supplement: Supplementary file 2 — Supplementary Material 2 [file 41598_2025_23191_MOESM2_ESM.pdf]

Supplementary Figure and Tables

Post-bottleneck increase in mitochondrial DNA diversity in Yaku sika deer (*Cervus nippon yakushimae*) on Yakushima Island, Japan

AUTHOR INFORMATION

*Yoshimi Agetsuma-Yanagihara[1] (Corresponding Author):

https://orcid.org/0000-0002-2672-7633

gg5762chacha@gmail.com

Takashi Hayakawa[2]:

https://orcid.org/0000-0002-1037-0350

Naoki Agetsuma[3]:

https://orcid.org/0000-0003-4093-5276

[1]Waku Doki Science Planning, Sapporo, Hokkaido 065-0016, Japan

[2]Faculty of Environmental Earth Science, Hokkaido University, Sapporo, Hokkaido 060-0810, Japan

[3]Field Science Center for Northern Biosphere, Hokkaido University, Sapporo, Hokkaido 060-0809, Japan

Supplementary Table S1 Haplotypes based on the mitochondrial DNA control region 894 base pairs from Yaku sika deer (*Cervus nippon yakushimae*) at Yakushima and Kuchinoerabujima.

Haplotype	Base number															N	Accession No. of DDBJ
	119	194	209	262	329	390	431	497	521	587	773	774	777	840	861		
Yk01	T	G	A	A	C	T	T	C	C	C	T	G	A	–	–	14	LC664029
Yk02	·	·	·	·	·	·	·	·	·	·	·	·	·	T	·	30	LC664030
Yk03	·	·	·	·	·	·	·	·	·	·	·	·	·	·	T	3	LC664031
Yk04	·	·	·	·	·	*C*	·	·	·	·	C	·	G	T	·	54	LC664032
Yk05	C	·	·	·	·	·	·	T	·	·	·	·	·	·	·	41	LC664033
Yk06	·	·	·	·	·	·	·	T	·	·	·	·	·	·	·	34	LC664034
Yk07	·	·	·	·	·	·	·	·	·	·	·	·	G	·	·	34	LC664035
Yk08	·	·	·	·	·	·	·	·	·	·	·	·	G	T	·	11	LC664036
Yk09	C	·	G	·	·	·	·	T	·	·	·	·	·	·	·	26	LC664037
Yk10	C	·	G	·	·	·	·	T	·	·	·	·	·	T	·	3	LC664038
Yk11	·	·	·	·	·	·	·	·	·	·	·	A	G	·	·	18	LC664039
Yk12	·	·	·	·	·	·	·	·	·	·	·	A	G	T	·	10	LC664040
Yk13	·	·	·	·	·	·	·	T	·	·	C	·	G	·	·	8	LC664041
Yk14	·	·	·	·	·	·	C	·	·	·	·	·	·	T	·	7	LC664042
Yk15	·	·	·	G	T	·	·	·	·	·	·	·	·	·	·	6	LC664043
Yk16	·	*A*	·	·	·	·	·	·	·	·	·	A	G	T	·	2	LC664044
Yk17	·	·	·	G	T	·	·	·	·	*T*	·	·	·	·	·	1	LC664045
Yk18	·	·	·	·	·	·	C	·	·	·	·	·	G	T	·	1	LC664046
Kc01	·	·	·	·	·	·	·	·	*T*	·	·	A	G	·	·	1	LC664047
Kc02	·	·	·	·	·	·	·	T	·	·	·	·	·	·	·	1	LC664048

The base numbering is based on the first base of the NCBI Accession No. AB279718.1.

Dots (･) indicate that the nucleotides are identical to the first sequence of each unit. A gap is indicated by –.

Italicized letters indicate a singleton site.

The read length of Kc02 is 841 bp.

Supplementary Table S2 The number of occurrences of each haplotype by Area.

Haplotype	Area								
	A	B	C	D	E	F	G	H	I
Yk01			3	6		2		1	2
Yk02	19				4	6		1	
Yk03					1			2	
Yk04	52						2		
Yk05	4	7	1	4	1	4	8		12
Yk06	15	2	2	4		3	8		
Yk07				9	8	2	1	13	1
Yk08	1				1			9	
Yk09		3	7				12		4
Yk10	3								
Yk11			3	5	6	1			3
Yk12	4	6							
Yk13			5	2					1
Yk14							7		
Yk15	6								
Yk16								2	
Yk17			1						
Yk18						1			

Supplementary Table S3 Genetic differentiation among nine areas (A–I) based on the mitochondrial DNA control region 894 base pairs analyzed using AMOVA.

Source of variance	df	Sum of squares	Variance components	Variation (%)	Fixation index
Among populations	8	92.67	0.34022 Va	26.27	Fst: 0.26267
Within populations	294	280.785	0.95505 Vb	73.73	$p < 0.0001$
Total	302	373.454	1.29528		

Supplementary Table S4 Mitochondrial DNA control region haplotypes of sika deer (*Cervus nippon*) in Japan used for network analysis.

Accession No. of NCBI	Locality		Haplotype name	Number of VNTRs 1)		Reference
				Randi	Nagata	
D50128	Hokkaido		Hka	6	7	[84]
D50129	Hokkaido		Hkb	6	7	[84]
AB210267.2	Hokkaido		–	6	7	[40]
AB794865	Iwate	Northern Honshu	Iwt1	5	6	[98]
AB772018	Iwate	Northern Honshu	Iwt2	6	7	[98]
AB772016	Miyagi	Northern Honshu	Osk1	6	7	[98]
AB772017	Miyagi	Northern Honshu	Osk2	6	7	[98]
AB247654	Chiba	Central Honshu	1a	5	6	[99]
AB247656	Chiba	Central Honshu	2a	6	7	[99]
LC481352	Yamanashi	Central Honshu	CN-2	10	11	[16]
AB772272	Shizuoka	Central Honshu	SHI5	7	8	[98]
LC333110	Gifu	Central Honshu	Cn08	6	7	[100]
AB248236.1	Mie	Southern Honshu	6Kii1	5	6	[13]
AB248237.1	Mie/Nara	Southern Honshu	6Kii2	5	6	[13]
AB248233.1	Nara	Southern Honshu	7Nra1	6	7	[13]
AB248234.1	Hyogo	Southern Honshu	7Hyg1	6	7	[13]
AB248235.1	Hyogo	Southern Honshu	7Hyg2	6	7	[13]
AB248238.1	Hyogo	Southern Honshu	6Hyg1	5	6	[13]
AB279713.1	Yamaguchi	Southern Honshu	4Ymg1	3	4	[14]
AB186350.1	Tokushima	Shikoku	4Esk2	3	4	[13]
AB186351.1	Tokushima	Shikoku	4Esk3	3	4	[13]
AB186352.1	Tokushima	Shikoku	4Esk4	3	4	[13]
AB757716.1	Tokushima	Shikoku	Tks1(4)	3	4	
AB186346.1	Tokushima/Kochi	Shikoku	7Esk1	6	7	[13]
AB186347.1	Tokushima/Kochi	Shikoku	6Esk1	5	6	[13]
AB186349.1	Tokushima/Kochi	Shikoku	4Esk1	3	4	[13]
AB186344.1	Tokushima/Kochi	Shikoku	9Esk1	8	9	[13]
AB186345.1	Kochi	Shikoku	8Esk1	7	8	[13]
AB757714.1	Kochi	Shikoku	Koc1(7)	6	7	
AB757715.1	Kochi	Shikoku	Koc2(4)	3	4	
AB279706.1	Ehime	Shikoku	4Wsk1	3	4	[14]
AB279707.1	Ehime	Shikoku	4Wsk2	3	4	[14]
AB279708.1	Ehime	Shikoku	4Wsk3	3	4	[14]
AB279709.1	Ehime	Shikoku	4Wsk4	3	4	[14]
AB279710.1	Ehime	Shikoku	4Wsk5	3	4	[14]
AB279711.1	Ehime	Shikoku	4Wsk6	3	4	[14]
AB279712.1	Ehime	Shikoku	4Wsk7	3	4	[14]

AB279714.1	Fukuoka		4Fko1	3	4	[14]
LC515803.1	Nagasaki		4SSB1(Hkj)	3	4	[101]
AB757720.1	Oita		Oit1(5)	4	5	
AB757721.1	Oita		Oit2(5)	4	5	
AB757722.1	Oita		Oit3(5)	4	5	
AB186348.1	Miyazaki		5Myz1	4	5	[14]
AB279723.1	Miyazaki		5Myz2	4	5	[14]
AB279724.1	Miyazaki		5Myz3	4	5	[14]
AB279725.1	Miyazaki		5Myz4	4	5	[14]
AB279715.1	Kagoshima		4Stm1	3	4	[14]
AB757717.1	Kagoshima		Kgo2(4)	3	4	
AB757718.1	Kagoshima		Kgo3(5)	4	5	
AB757719.1	Kagoshima		Kgo4(6)	5	6	
AB871968.1	Kagoshima/Miyazaki		KY4A	3	4	[69]
AB871969.1	Kumamoto		KY4B	3	4	[69]
AB871970.1	Oita	Mainland	KY4C	3	4	[69]
AB871971.1	Oita	Kyushu	KY4D	3	4	[69]
AB871972.1	Oita		KY4F	3	4	[69]
AB871969.1	Oita		KY4G	3	4	[69]
AB871974.1	Oita		KY4H	3	4	[69]
AB871975.1	Oita		KY4I	3	4	[69]
AB871976.1	Oita		KY4J	3	4	[69]
AB871977.1	Oita		KY4K	3	4	[69]
AB871978.1	Kagoshima		KY5A	4	5	[69]
AB871979.1	Kagoshima		KY5B	4	5	[69]
AB871980.1	Kagoshima/Miyazaki		KY5C	4	5	[69]
AB871981.1	Kumamoto		KY5D	4	5	[69]
AB871982.1	Oita		KY5E	4	5	[69]
AB871983.1	Oita		KY5F	4	5	[69]
AB871984.1	Oita		KY5G	4	5	[69]
AB871985.1	Oita		KY5H	4	5	[69]
AB871986.1	Oita		KY5I	4	5	[69]
AB871987.1	Oita		KY5J	4	5	[69]
AB871988.1	Kagoshima		KY6A	5	6	[69]
AB871989.1	Kumamoto		KY6B	5	6	[69]
AB279714.1	Tsushima		4Tsm1	3	4	[14]
AB279717.1	Goto		4Gto1	3	4	[14]
AB279718.1	Tanegashima		4Tng1	3	4	[14]
AB279719.1	Tanegashima		4Tna2	3	4	[14]
AB757723.1	Tanegashima		Tng3(4)	3	4	
AB757724.1	Tanegashima		Tng4(4)	3	4	

LC712848.1	Mageshima	MG301	3	4	[68]
LC712849.1	Mageshima	MG302	3	4	[68]
LC712850.1	Mageshima	MG304	3	4	[68]
LC712851.1	Mageshima	MG310	3	4	[68]
LC712852.1	Mageshima	MG312	3	4	[68]
AB218689.1	Yakushima	–	3	4	[40]
LC664029	Yakushima	Yk01	3	4	This study
LC664030	Yakushima	Yk02	3	4	This study
LC664031	Yakushima	Yk03	3	4	This study
LC664032	Yakushima	Yk04	3	4	This study
LC664033	Yakushima	Yk05	3	4	This study
LC664034	Yakushima	Yk06	3	4	This study
LC664035	Yakushima	Yk07	3	4	This study
LC664036	Yakushima	Yk08	3	4	This study
LC664037	Yakushima	Yk09	3	4	This study
LC664038	Yakushima	Yk10	3	4	This study
LC664039	Yakushima	Yk11	3	4	This study
LC664040	Yakushima	Yk12	3	4	This study
LC664041	Yakushima	Yk13	3	4	This study
LC664042	Yakushima	Yk14	3	4	This study
LC664043	Yakushima	Yk15	3	4	This study
LC664044	Yakushima	Yk14	3	4	This study
LC664045	Yakushima	Yk17	3	4	This study
LC664046	Yakushima	Yk18	3	4	This study
LC664047	Kuchinoerabujima	Kc01	3	4	This study
LC664048 2)	Kuchinoerabujima	Kc02	3	4	This study
LC712853.1	Kuchinoerabujima	KUE201	3	4	[68]
LC712854.1	Kuchinoerabujima	KUE202	3	4	[68]
LC712855.1	Kuchinoerabujima	KUE203	3	4	[68]
LC712856.1	Kuchinoerabujima	KUE205	3	4	[68]
LC712857.1	Kuchinoerabujima	KUE206	3	4	[68]
AB871966.1	Kerama	KER4	3	4	[69]
AB871967.1	Kerama	KER5	4	5	[69]

1) VNTR counts based on Randi [63] and Nagata [12], respectively. Randi's count includes the original sequence.

2) Excluded from haplotype network and molecular phylogenetic analyses due to short read length.
